# Peer Review of “Representing Physician Suicide Claims as Nanopublications: Proof-of-Concept Study Creating Claim Networks”

**DOI:** 10.2196/39886

**Published:** 2022-07-01

**Authors:** Eric C Chan

**Affiliations:** 1 Department of Psychiatry University of Calgary Calgary, AB Canada

**Keywords:** physician suicide, suicide, suicide prevention, physician well-being, physician mental health, nanopublication, physician, doctor, mental health, semantic publishing, bibliometrics, claim network, information distortion, misinformation

*This is a peer-review report submitted for the paper “Representing Physician Suicide Claims as Nanopublications: Proof-of-Concept Study Creating Claim Networks.”*

## Round 1 Review

### General Comments

This study [1] describes the use of nanopublications as a means to create a citation network of claims. The authors suggest that this approach allows for verification of claims in scientific literature. In the case of this particular article, the authors describe a process in which nanopublications are created from assertions of physician suicide incidence and describe their findings. Notably, the authors report that “the network is not fully connected,” “no single primary source of the claim could be identified,” and “all end-point citations either had a claim with no further citation, had no apparent claim, or could not be accessed to verify the claim.”

I believe this work is important for the methods used and the purpose of the study more than it is for the actual finding itself (which is also important). Properly implemented, the approach used could be very important in improving the validity of claims cited in scientific literature. As demonstrated in this study, it is important that assertions be verifiable in order to prevent the propagation of misinformation or distorted information. The propagation of misinformation can impact future work, as the assertions may influence the way future researchers pursue investigation. Furthermore, misinformation or inaccurate information in peer-reviewed literature can negatively impact the perceived integrity of the scientific process. As such, I believe the methods used by the authors deserve attention but should also be examined carefully to ensure the way in which this approach is implemented is thorough and can accurately identify the primary source of claims if possible.

The authors do an excellent job of describing the purpose of their work and provide the spreadsheet used to create the nanopublication index. This is helpful in evaluating the work and ensuring accuracy. Given the importance of this work, one aspect of the methodology is unclear and, in my opinion, should be made clear before the article could be considered suitable for publication.

### Specific Comments

#### Major Comments

The process to determine how an assertion was cited (if at all) is unclear. Optimally, any statement providing quantitative information, such as the one investigated in this study, should be directly followed by the relevant citation. This is not always the case, however, especially when multiple statements are made based on the same source and especially if they build on each other. If the authors only consider citations immediately following the assertion, they may have missed the reference provided shortly prior, or at the end of the paragraph. It would be helpful if the authors provided additional detail on this process, so that this process can be applied more consistently by other research teams using this approach.

While I commented on the process in which they determine which citations to evaluate, I have also examined the articles in Figure 2 for which no reference was provided. The only potentially missed reference was a 1977 JAMA article by Sargent et al [2]. Regarding that paper, the relevant statistic (300-400 physicians per year) is not mentioned anywhere. Overall, I am not concerned about the thoroughness of the process used but advise that the exact details be included in the methods.

#### Minor Comments

Figure 2 appears to have some errors (there may be others I have not noticed; I suggest the authors review the entire figure for accuracy):

- The nanopublication links for Withy et al [3] and Anzia et al [4] are identical; it appears the link for Anzia (2016) in the figure is incorrect when compared to the excel file.
- The year of publication for what appears to be Andrew & Brenner (2018) in the excel file is listed as 2015.

## Round 2 Review

### General Comments

The authors appear to have addressed many of the concerns raised by me and by other reviewers, but some comments still have not been addressed satisfactorily. I do feel this work is important, but the below comments should be addressed before publication.

### Specific Comments

#### Major Comments

1. Other reviewers brought up the statement “Additional articles published between August 2019 and March 2020 have been identified and manually added to the article set used for this study.” While I believe the authors clarified a separate concern raised by one of the authors, this statement requires additional clarification. It is unclear how those articles were identified, and this should be explained.

2. The authors appear to have misunderstood my following comment: “The process to determine how an assertion was cited (if at all) is unclear. Optimally, any statement providing quantitative information, such as the one investigated in this study, should be directly followed by the relevant citation. This is not always the case, however, especially when multiple statements are made based on the same source and especially if they build on each other. If the authors only consider citations immediately following the assertion, they may have missed the reference provided shortly prior, or at the end of the paragraph. It would be helpful if the authors provided additional detail on this process, so that this process can be applied more consistently by other research teams using this approach”.

To clarify, I would like more details on how it was determined which sources were cited to support a claim. For example, if a paper contained a paragraph with the assertion in question, it may not always have the relevant citation at the end of the statement. Take the following hypothetical statement (not from any actual paper, but for illustrative purposes):

“Physician suicide remains an important topic related to the health status of the workforce, but previous studies indicate that there are little data on the subject in the scientific literature [1-4]. 300 to 400 US physicians die by suicide annually, and a recent economic analysis estimates that physician suicide results in the loss of US $XXXXX per year from the American health care system [5]. Consequently, physician suicide is the *Y*th cause of death among physicians [6].”

The “300 to 400” physician number should be found in reference 5, but that is not always the case, especially if later edits were made and the change was not noticed. Sometimes, there is no citation “5,” and the statistic is derived from references 1-4 or 6. While this is obviously not good practice, it does occur in scientific papers occasionally and not infrequently in other types of publications (while I cannot remember for sure, I believe one of the studies cited is missing a citation similar to 5). I would like to clarify if the authors attempted to account for other proximal references, which is different from snowballing, but arguably may catch sources that otherwise could be missed.
